# ATF3-based peripheral neural tract tracing

**DOI:** 10.3389/fnana.2026.1842056

**Published:** 2026-05-15

**Authors:** Laurent Gautron

**Affiliations:** Department of Internal Medicine and Center for Hypothalamic Research, UT Southwestern Medical Center, Dallas, TX, United States

**Keywords:** autonomic nervous system, axotomy, tracers, transcription factor, vagus nerve

## Abstract

Defining neural connectivity between peripheral organs and the nervous system remains a fundamental challenge. While neural tract tracing techniques are well established and highly effective in the central nervous system, their application in peripheral tissues—particularly through retrograde approaches—continues to generate controversy. This article highlights an alternative approach based on the neuronal injury response. Specifically, I discuss the use of activating transcription factor 3 (ATF3), a molecular marker robustly upregulated in neurons following axonal injury, as an indirect means of identifying organ-specific innervation. By integrating classical and injury-induced approaches, this framework may help resolve longstanding debates regarding visceral innervation and improve the reliability of circuit-mapping strategies in the peripheral nervous system.

## Introduction

“*Our experiments show therefore that the spleen receives neither excitatory neither inhibitory fibers from the vagus, but is innervated entirely through the sympathetic*” (Magnus and Schafer, 1901).

The words of Magnus and Schäfer, written in 1901, highlight a longstanding debate regarding the innervation of peripheral organs. At the time, investigators could not agree on whether the spleen receives input from the vagus nerve. Based on their physiological experiments, Magnus and Schäfer concluded that reports of vagal innervation of the spleen were likely artifacts. Surprisingly, the extent of vagal innervation remains contested even today (Berthoud et al., 2006; Trevizan-Bau and McAllen, 2024; Berthoud et al., 2025). This ongoing uncertainty underscores the need for reliable tracing approaches to clarify the neural connections of the peripheral organs. The central question is whether specific neuronal populations are truly connected to organs or visceral tissues.

Establishing definitive connections between neurons and target organs ideally relies on neural tract tracing techniques. These methods are well established, technologically advanced, and have been widely used for decades (Luo et al., 2018). While their application in the central nervous system (CNS) is relatively straightforward, challenges arise when studying peripheral neurons, including sensory and motor populations. Anterograde tracing—achieved by delivering tracers or viral vectors to sites containing neuronal somata (such as ganglia or central nuclei)—is generally reliable and informative (Berthoud et al., 1991). In contrast, retrograde tracing from peripheral organs remains controversial.

**Figure 1:**
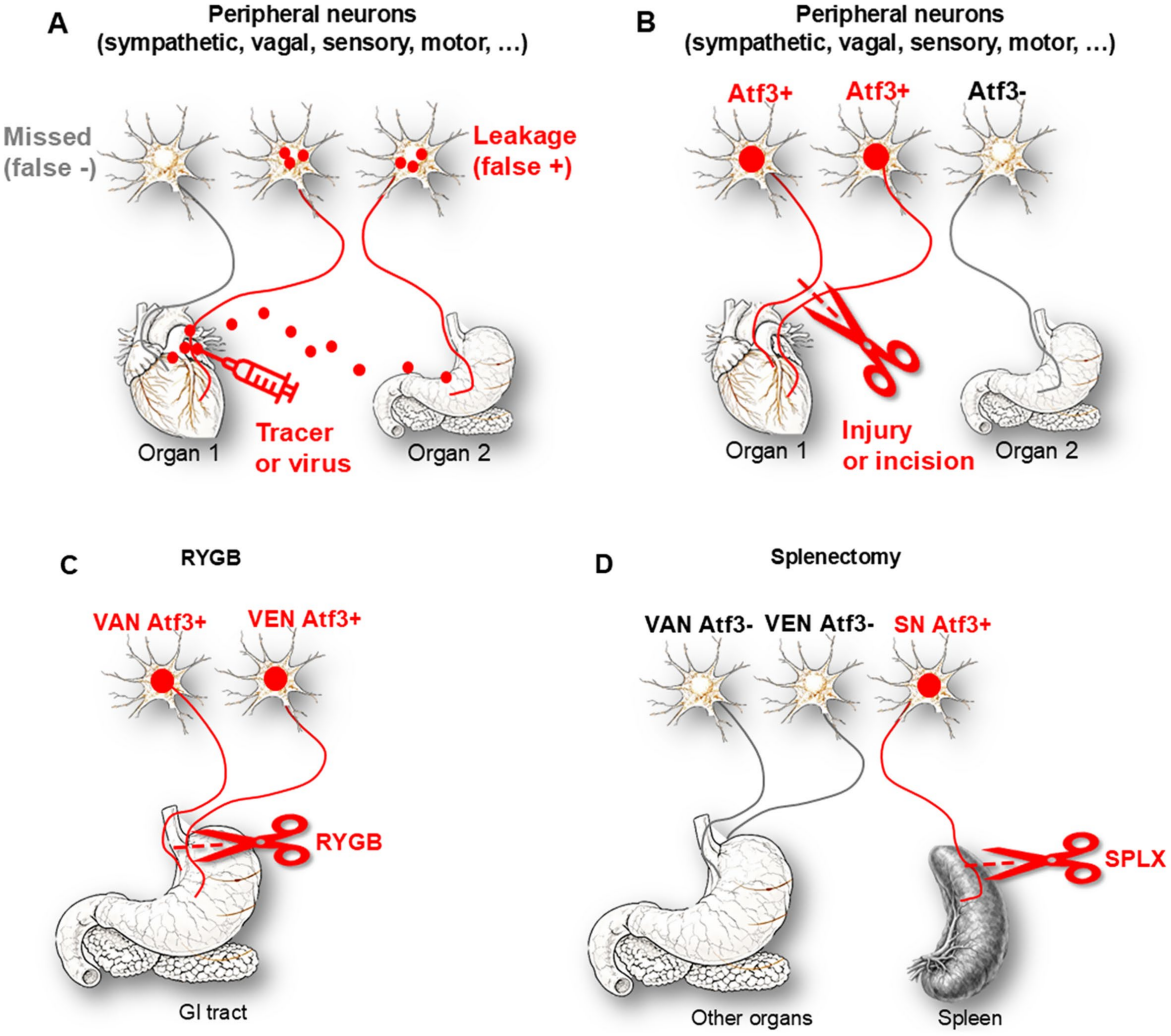
Basic principles associated with peripheral neural tract tracing. **(A)** Conventional approaches involve the injection of a small volume of a neurotropic tracer or viral vector into a specific organ (red dots). Major limitations include incomplete tracer diffusion, which may lead to suboptimal labeling of projecting neurons, and unintended leakage of tracer into the bloodstream, which can result in labeling of neurons innervating other organs and generate false-positive signals. **(B)** ATF3-based tracing exploits a characteristic property of peripheral neurons: following injury to their peripheral axons, these neurons strongly upregulate the expression of the transcription factor ATF3. This injury-induced expression can be detected by immunohistochemistry or *in situ* hybridization (red nuclei). Leveraging this response enables the identification and mapping of neurons that innervate a targeted peripheral tissue with improved anatomical specificity. **(C)** Example of ATF3-based tracing following surgery. After Roux-en-Y gastric bypass (RYGB), mice exhibit specific *Atf3* expression in neurons connected to the gastrointestinal (GI) tract that are affected by the procedure (Merchant et al., 2025). **(D)** Example of ATF3-based tracing of neurons connected to the mouse spleen. Following splenectomy (SPLX), neurons innervating the spleen—such as sympathetic neurons—upregulate *Atf3*, whereas neurons in the vagus nerve do not (Khan et al., 2026). RYGB, Roux-en-Y gastric bypass; SPLX, splenectomy; VAN, vagal afferent neurons; VEN, vagal efferent neurons; SN, sympathetic neurons.

Retrograde approaches typically involve injecting a neurotropic tracer or a viral vector expressing a fluorescent reporter into the target organ using a needle or glass micropipette (Figure 1A). Once the tracer or virus is taken up by nerve terminals and transported back to ganglia and nerve centers, it can be detected in neurons connected to the specific organ (de Beer, 2022; Sterner et al., 1985; Cano et al., 2001). However, when tracers or viral vectors are injected into peripheral tissues, leakage is difficult to avoid and varies depending on organ type, injection volume, and local vascularization. If the injected volume of tracer is too small, then many neurons are going to be missed (Figure 1A). However, when injecting large volumes, uptake into the vasculature can result in off-target labeling far from the intended injection site (Fox and Powley, 1989). Unlike intracerebral injections, where tracers typically remain spatially confined, peripheral injections often produce inaccurate connectivity maps. Numerous experts have called for increased attention to these recurring issues (Berthoud et al., 2006; Trevizan-Bau and McAllen, 2024; Berthoud et al., 2025).

### ATF3-based tracing

An alternative retrograde strategy leverages the intrinsic neuronal injury response rather than relying on exogenous tracer administration. The concept of “retrograde degeneration” is not new; it was already explored in the early 20th century to identify neuronal connections in various nerves, including the vagus nerve (Molhant, 1911). However, these early approaches relied on non-molecular techniques, primarily the histological identification of stained neurons exhibiting features of degeneration. As a result, such methods were technically challenging, variable, and often difficult to interpret. Over time, they were progressively abandoned in favor of tracer-based techniques, such as those discussed above.

Here, I propose a method based on the same principle as “retrograde degeneration” but relying on molecular histology. Following axotomy, peripheral neurons robustly upregulate activating transcription factor 3 (ATF3), a well-established marker of neuronal injury (Braz and Basbaum, 2010; Linda et al., 2011; Braz et al., 2011). I argue that ATF3-based tracing represents a valid method for mapping neuronal connectivity under specific conditions. This approach is applicable to a wide range of peripheral neuron types and target organs. Injury can be induced by transecting a nerve or one of its branches, damaging a target organ at a defined location, or even removing an organ entirely (if feasible) (Figure 1B). This property has been recently used by us to map connections (or their absence), such as those between the nodose ganglion and the stomach, and between the brainstem and the spleen. For example, after Roux-en-Y gastric bypass (RYGB), mice exhibit specific *Atf3* expression in a subset of nodose ganglion neurons connected to the gastrointestinal (GI) tract that are damage during the surgery (Merchant et al., 2025) (Figure 1C). Additionally, when the spleen is removed from a mouse, *Atf3* expression—normally low or absent in peripheral neurons—becomes detectable in neurons connected to the spleen. In our hands, *Atf3* expression detected by RNAscope was visible in celiac ganglion neurons but not in vagal neurons (Khan et al., 2026) (Figure 1D).

This approach offers several advantages. It eliminates the need for solution injections, thereby avoiding leakage-related artifacts and reducing experimental variability. In principle, this strategy is feasible across animal species, and current evidence suggests that many classes of peripheral neurons upregulate ATF3 following injury. Most importantly, this method avoids biases inherent to tracers or viral vectors.

However, several caveats must be considered. Not all nerves can be selectively lesioned—particularly very small branches—and some organs cannot be injured or removed without compromising animal survival. Additionally, ATF3-positive neurons cannot be phenotyped in their native transcriptional state, as injury induces profound gene expression changes (Renthal et al., 2020). For downstream characterization, permanent genetic labeling strategies (e.g., GFP-based approaches) are therefore required. Further studies are needed to determine the optimal time window for detecting ATF3 expression after injury, to compare ATF3 immunohistochemistry with *Atf3* mRNA detection, and to assess whether all neurons produce ATF3 equally in response to injury.

## Discussion

Our focus is not on ATF3 as a molecule involved in regeneration or survival per se, but rather on its potential utility as a marker. ATF3, which is induced in injured peripheral neurons, can be used to identify and trace neural pathways between organs and the peripheral nervous system.

By analogy, early immediate genes (e.g., Fos) have been widely used as markers of neuronal activation in the past. Likewise, I suggest that ATF3 could potentially serve as a marker for tracing pathways, provided that experimental designs allow visualization of ATF3 expression following specific peripheral lesions.

ATF3-based tracing represents a powerful complementary strategy for peripheral retrograde mapping. Although not universally applicable, it provides a compelling alternative in contexts where conventional peripheral tracer or viral approaches are confounded by leakage or missed injections. Given that off-target spread in peripheral tracing studies is likely underestimated, greater consideration of injury-induced labeling strategies is warranted.

## Data Availability

The original contributions presented in the study are included in the article/supplementary material, further inquiries can be directed to the corresponding author.
